# Enhancing early detection of cognitive decline in the elderly: a comparative study utilizing large language models in clinical notes

**DOI:** 10.1016/j.ebiom.2024.105401

**Published:** 2024-10-12

**Authors:** Xinsong Du, John Novoa-Laurentiev, Joseph M. Plasek, Ya-Wen Chuang, Liqin Wang, Gad A. Marshall, Stephanie K. Mueller, Frank Chang, Surabhi Datta, Hunki Paek, Bin Lin, Qiang Wei, Xiaoyan Wang, Jingqi Wang, Hao Ding, Frank J. Manion, Jingcheng Du, David W. Bates, Li Zhou

**Affiliations:** a Division of General Internal Medicine and Primary Care, Brigham and Women's Hospital, Boston, MA, 02115, USA; b Department of Medicine, Harvard Medical School, Boston, MA, 02115, USA; c Division of Nephrology, Taichung Veterans General Hospital, Taichung, 407219, Taiwan; d Department of Post-Baccalaureate Medicine, College of Medicine, National Chung Hsing University, Taichung, 402202, Taiwan; e School of Medicine, College of Medicine, China Medical University, Taichung, 406040, Taiwan; f Department of Neurology, Brigham and Women's Hospital, Boston, MA, 02115, USA; g Intelligent Medical Objects, Rosemont, Illinois, 60018, USA

**Keywords:** Cognitive dysfunction, Natural language processing, Neurobehavioral manifestations, Electronic health records, Early diagnosis, Alzheimer disease, Dementia

## Abstract

**Background:**

Large language models (LLMs) have shown promising performance in various healthcare domains, but their effectiveness in identifying specific clinical conditions in real medical records is less explored. This study evaluates LLMs for detecting signs of cognitive decline in real electronic health record (EHR) clinical notes, comparing their error profiles with traditional models. The insights gained will inform strategies for performance enhancement.

**Methods:**

This study, conducted at Mass General Brigham in Boston, MA, analysed clinical notes from the four years prior to a 2019 diagnosis of mild cognitive impairment in patients aged 50 and older. We developed prompts for two LLMs, Llama 2 and GPT-4, on Health Insurance Portability and Accountability Act (HIPAA)-compliant cloud-computing platforms using multiple approaches (e.g., hard prompting, retrieval augmented generation, and error analysis-based instructions) to select the optimal LLM-based method. Baseline models included a hierarchical attention-based neural network and XGBoost. Subsequently, we constructed an ensemble of the three models using a majority vote approach. Confusion-matrix-based scores were used for model evaluation.

**Findings:**

We used a randomly annotated sample of 4949 note sections from 1969 patients (women: 1046 [53.1%]; age: mean, 76.0 [SD, 13.3] years), filtered with keywords related to cognitive functions, for model development. For testing, a random annotated sample of 1996 note sections from 1161 patients (women: 619 [53.3%]; age: mean, 76.5 [SD, 10.2] years) without keyword filtering was utilised. GPT-4 demonstrated superior accuracy and efficiency compared to Llama 2, but did not outperform traditional models. The ensemble model outperformed the individual models in terms of all evaluation metrics with statistical significance (p < 0.01), achieving a precision of 90.2% [95% CI: 81.9%–96.8%], a recall of 94.2% [95% CI: 87.9%–98.7%], and an F1-score of 92.1% [95% CI: 86.8%–96.4%]. Notably, the ensemble model showed a significant improvement in precision, increasing from a range of 70%–79% to above 90%, compared to the best-performing single model. Error analysis revealed that 63 samples were incorrectly predicted by at least one model; however, only 2 cases (3.2%) were mutual errors across all models, indicating diverse error profiles among them.

**Interpretation:**

LLMs and traditional machine learning models trained using local EHR data exhibited diverse error profiles. The ensemble of these models was found to be complementary, enhancing diagnostic performance. Future research should investigate integrating LLMs with smaller, localised models and incorporating medical data and domain knowledge to enhance performance on specific tasks.

**Funding:**

This research was supported by the National Institute on Aging grants (R44AG081006, R01AG080429) and National Library of Medicine grant (R01LM014239).

**Research in context**

**Evidence before this study**

We searched PubMed and Web of Science for publications from database inception to 19 May 2023, using a broad range of keywords related to generative large language models (LLMs), including chatbot, generative artificial intelligence (AI), and commonly available LLMs. Among the 6332 distinct articles retrieved, several knowledge gaps were identified: 1) no studies addressed the early detection of cognitive decline using LLMs; 2) only four studies analysed real electronic health record (EHR) data with LLMs in HIPAA-compliant cloud computing platforms; 3) no studies compared LLMs with traditional AI approaches, such as conventional deep learning and machine learning models.

**Added value of this study**

This study conducted a comparative analysis of the performance of LLMs against other deep learning and traditional machine learning methods using real EHR data. The study included both open-source and proprietary LLMs and utilised 6945 annotated clinical notes from real EHR data. Our main scientific contributions are as follows: 1) We developed and evaluated advanced prompting strategies, including few-shot, retrieval augmented generation (RAG), and error analysis-based instructions, finding that adding error-analysis-based instructions led to the best performance. 2) We observed that the best-performing LLM with an optimised prompting strategy did not outperform traditional AI methods trained with local and domain-specific EHR data. 3) A comprehensive analysis revealed that the error profiles of LLMs trained with a general domain corpus, locally trained deep learning models, and machine learning models are markedly different; combining them into an ensemble significantly boosted performance.

**Implications of all the available evidence**

Our findings underscore that general-domain LLMs require further refinement for clinical decision-support tasks and applications. Future research should explore strategies for prompting, model fine-tuning, and integrating LLMs with smaller, localised models and knowledge bases to enhance task-specific performance.

## Introduction

Large Language Models (LLMs), neural models with billions of parameters trained on extensive and diverse text corpora, have demonstrated remarkable capabilities in clinical language understanding tasks.^1^, ^2^, ^3^, ^4^, ^5^ They offer distinct advantages over traditional rule-based and machine learning approaches, which are often trained from scratch on narrower clinical datasets.^6^, ^7^, ^8^ Previous studies have shown that LLMs achieve impressive performance in a variety of clinical natural language processing (NLP) tasks, such as question answering, named entity recognition, and information extraction.^1^^,^^2^ However, the effectiveness of LLMs in identifying specific clinical conditions within real medical records remains underexplored. Their lack of explicit training on specific medical records may impact their accuracy.^9^ This study aims to evaluate the performance of LLMs in detecting signs of cognitive decline within clinical notes, using this as a case study to explore their effectiveness and compare their error profiles with those of traditional models trained on domain-specific corpora. The insights gained will inform strategies for further enhancement.

Alzheimer's disease (AD) and related dementias (ADRD) affect millions of Americans,^10^ significantly reducing patient quality of life and imposing substantial emotional and financial burdens,^11^ with care costs projected to reach $1.1 trillion by 2050.^12^ Existing treatments offer only temporary relief,^13^ highlighting the urgent need for breakthroughs in AD/ADRD therapy.^14^ Timely detection of cognitive decline can facilitate early interventions and support involvement in clinical trials for AD/ADRD.^15^, ^16^, ^17^, ^18^ Electronic health records (EHRs), particularly clinical notes, are crucial resources for identifying early indicators of disease, yet traditional diagnostic tools and variability in screening practices complicate detection.^19^, ^20^, ^21^, ^22^ NLP provides a promising solution by efficiently analysing large datasets and identifying subtle signs of decline that traditional diagnostics may miss.^23^ Although studies have been conducted to identify cognitive decline using NLP,^7^^,^^24^, ^25^, ^26^ the effectiveness of LLMs in identifying cognitive decline through EHRs remains under-explored.

This research utilises LLMs within HIPAA-compliant computing environments for a pioneering exploration of EHR note analysis for cognitive decline detection. It evaluates the effectiveness and interpretability of LLMs compared to conventional machine learning methods and examines the synergy between LLMs and machine learning to enhance diagnostic accuracy. To the best of our knowledge, this initiative employs LLMs in detecting cognitive decline from clinical notes, representing a valuable innovation and contribution to biomedical informatics.

## Methods

### Ethics

The study received approval from the MGB Institutional Review Board (Protocol #:2022P002987), with a waiver of informed consent for study participants due to the secondary use of EHR data.

### Setting and datasets

This study was conducted at Mass General Brigham (MGB), a large integrated healthcare system in Massachusetts, which has established secure, HIPAA-compliant cloud environments for deploying and evaluating LLMs with actual EHR data. Two LLMs were tested: the proprietary GPT-4^1^ via Microsoft Azure OpenAI Service API, and the open-source Llama 2 (13B)^2^ via an Amazon Elastic Compute Cloud (EC2) instance. Details on the cloud environments are provided in Supplementary Material Section 1 and Table S1. We followed the Transparent Reporting of a Multivariable Prediction Model for Individual Prognosis or Diagnosis (TRIPOD) guidelines for designing and reporting this study.^27^

We utilised the same definition of cognitive decline and annotated datasets from a previous study.^19^ The determination of cognitive decline aimed to identify patients at any stage, from Subjective Cognitive Decline (SCD) to Mild Cognitive Impairment (MCI) to dementia. Cognitive decline can be identified through various indicators, including the mention of cognitive concerns, symptoms (e.g., memory loss), diagnoses (e.g., MCI, Alzheimer's Disease dementia), cognitive assessments (e.g., Mini-Cog) — even if patients show normal performance but have documented cognitive concerns — or cognitive-related therapies or treatments (e.g., cognitive-linguistic therapy). Our focus was on progressive cognitive decline, which is likely to be consistent with or lead to MCI. Cases were considered negative for cognitive decline if they were less likely to be progressive (e.g., cognitive function improvement), transient (e.g., temporary forgetfulness or occasional memory loss due to medication such as codeine), or reversible (e.g., cognitive impairment shortly after events like surgery, injury, or stroke). Additionally, sections of notes were labelled as negative when records indicated broader or uncertain signs of cognitive decline.

To create the annotated dataset, three annotators received training from subject matter experts to label sections of clinical notes for cognitive decline. Initially, each annotator independently labelled 150 sections, with any conflicts resolved through discussion with the subject matter experts. In a subsequent dataset of 50 sections, the three annotators achieved a high level of agreement, evidenced by a Fleiss κ value of 0.83. Following this, additional sections were annotated by one of the three annotators. Any cases with uncertain labelling were resolved by consulting the subject matter experts.

The annotated datasets comprised sections of clinical notes from the four years prior to the initial diagnosis of mild cognitive impairment (MCI, ICD-10-CM code G31.84) in 2019, for patients aged 50 years or older.^19^ Due to the low positive case rate across the sections, we used a list of expert-curated keywords (Table 1) to screen for sections likely indicating cognitive decline.

**Table 1:** Keywords contributing to the identification of positive cognitive decline cases, curated by domain experts and extracted from AI models.^a^

Model	Keywords
Expert Curated	Memory, agitat-, alter, alzheimer, attention, cognit-, confus-, decline, delirium, dementia, difficult, disorientation, drive, evaluat-, exam, forget-, function, impairment, loss, mental, mild, mmse, moca, montreal, mood, neuro-, orientation, psych-, question, recall, remember, score, sleep, speech, word, worse
XGBoost	Cognitive, dementia, forgetful, memory
Attention-Based DNN	BNT, FTD, HOH, LBD, MCI, MMSE, Memory, MoCA, abstraction, aforementioned, age, alzheimer, alzheimers, amnestic, amyloid, aphasa, attention, attentional, auditory, behavioral, category, challenges, clock, cog, cognition, cognitive, dementia, comprehension, correctly, cube, decline, deficit, deficits, delay, delayed, developmental, died, difficulties, encoding, errors, executive, expressive, falls, finding, fluency, forgetful, forgetfulness, forgets, forgetting, frailty, functional, functioning, global, hearing, immediate, impaired, impairment, insight, items, language, lapses, learning, linguistic, moderately, multidomain, names, naming, neurocognitive, neurodegenerative, perseveration, personality, phonemic, processing, recall, recalling, remember, remembering, repetition, retrieval, semantic, solving, span, spatial, speech, trails, visual, visuospatial, word, words, years
GPT4-8K	Altered mental status, Aricept, Impaired, MCI, MOCA, altered mental status, anxiety, attention, battery of neuropsychological tests, cognition, cognitive changes, cognitive concerns, cognitive decline, cognitive deficits, cognitive difficulties, cognitive impairment, cognitive issues, cognitive symptoms, cognitive-linguistic therapy, concerns, confused, confusion, current level of cognitive functioning, deficits, delayed recall, delirium, dementia, donepezil, executive function, executive functioning, forgetful, forgetfulness, language, major neurocognitive disorder, memory, memory complaints, memory concerns, memory difficulties, memory impairment, memory issues, memory loss, memory problems, mild cognitive impairment, mild dementia, mild neurocognitive disorder, neurocognitive disorder, neurocognitive status, neurodegenerative process, neuropsych testing, neuropsychological evaluation, neuropsychological testing, neuropsychological tests, poor safety awareness, problem solving, processing speed, short term memory loss, vascular dementia, verbal fluency, weakness, word finding difficulties, word-finding difficulties, working memory

a The table lists keywords that had a high frequency of appearance in the LLM's output (i.e., the number of appearances is higher than the average appearance time plus two standard deviations); keywords whose attention weights (from the attention-based DNN) exceeded the mean weights plus two standard deviations within individual sections; and keywords with an information gain (XGBoost) higher than the average value plus two standard deviations. We found that keywords identified by AI models could significantly enrich the expert-curated keyword set. Notably, only GPT-4 identified keywords related to medications for cognitive decline.

### LLMs and prompting methods

**Fig. 1:**
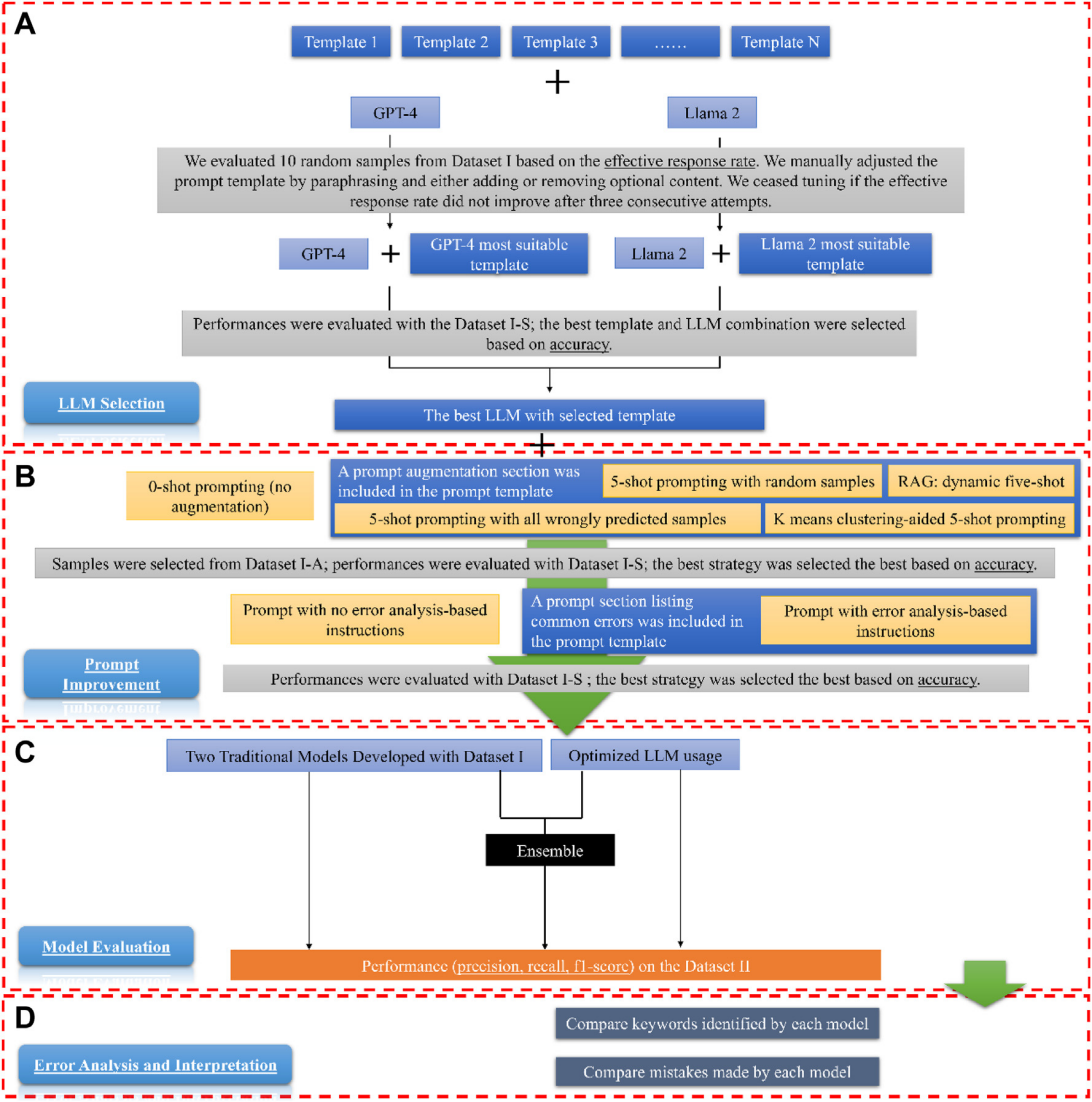
Study Design Overview. The workflow consists of four parts: A) LLM Selection: We fed prompts, which contain task descriptions and may also include additional task guidance as illustrated in Supplementary Figure S1, to GPT-4 and Llama 2 separately. We used 10 random samples from Dataset I to select the most suitable template for each LLM. During this process, if the effective response rate (i.e., the rate at which the response answered the questions in the prompt) was not 100%, we manually adjusted the template for each model. If the effective response rate did not improve after three consecutive attempts, we ceased tuning and used the template that led to the highest effective response rate. We then selected the best LLM based on their accuracy on Dataset I-S. B) Prompt Improvement: This step includes two sub-steps: prompt augmentation and adding error analysis-based instructions. During prompt augmentation, we tested whether five-shot prompting could improve accuracy. We then assessed whether incorporating instructions following an error analysis of the LLM's output on Dataset I–S could enhance accuracy. C) Model Evaluation: We evaluated the selected LLM and two traditional machine learning models. We also tested the performance of an ensemble model, which took the majority vote of the three models as the predictive label. D) Interpretation and Error Analysis: For interpretation, we examined and compared keywords used by each model for prediction, in conjunction with those curated by domain experts. Lastly, we analysed and compared errors made by each model.

Fig. 1 (areas A and B) illustrates the two-step prompt engineering process: LLM selection and prompt improvement. Following previous studies, we divided the prompt into sections.^28^ Supplementary Figure S1 shows the prompt structure, which includes a required task description and optional sections for prompt augmentation, error analysis-based instructions, and additional task guidance. We were cautious about the potential impact of longer prompts, which might overwhelm the model, negatively affecting performance, response speed, and cost efficiency.^29^, ^30^, ^31^ Therefore, as an initial step, we evaluated the performance of the two LLMs using manual template engineering and a smaller sample size. This approach enabled us to select the superior model and its corresponding prompt template for further analysis.^32^ The selection criterion was $ccuracy=\frac{true\;positive\;+\;true\;negative}{all\;cases}$, based on Dataset I–S. Using this metric and guided by the accuracy from Dataset I–S, we explored whether common prompt augmentation methods, including both hard prompting and retrieval augmented generation (RAG)^32^ and error analysis-based instructions^33^ could improve model performance. To ensure control over randomness and creativity, we adjusted the LLM's temperature hyperparameter to 0, providing a deterministic solution.^33^

#### LLMs comparison and selection

We utilised an intuitive manual template engineering approach to fine-tune the task description and additional task guidance for each LLM.^32^ During the iterative refinement process, we focused on the following task descriptions for each LLM: 1) identifying evidence of cognitive decline in clinical notes; 2) displaying which keywords in the clinical notes informed its judgment on the assigned task; and 3) requiring LLM responses in JavaScript Object Notation (JSON) format to facilitate straightforward parsing. Furthermore, we explored the possibility of adding additional task guidance to assist the LLM in its reasoning and enhance performance. Specifically, we considered two approaches: 1) requesting the LLM to provide reasoning for its judgments, and 2) incorporating our definition of cognitive decline directly into the prompt.

##### Manual template engineering

We fed each prompt to GPT-4 and Llama 2 separately. The responses from these LLM were classified into three categories (Supplementary Table S2): 1) effective and parseable: the LLM's response provides answers to both questions—whether cognitive decline was identified and which keywords were used for the decision—using a standard JSON format; 2) effective but not parseable: the LLM's response answers both questions, but does not adhere to the standard JSON format; 3) not effective: the LLM's response fails to answer either of the two questions. We assessed model effectiveness using 10 random samples from Dataset I. Our observations indicated that this sample size was sufficient for a meaningful comparison. If the effective response rate did not reach 100%, we manually adjusted the prompt template by paraphrasing or modifying optional content. This tuning process continued until no further improvement in the effective response rate was achieved after three consecutive attempts. Finally, we selected the prompt template that yielded the highest effective response rate for GPT-4 and Llama 2 separately.

##### Performance comparison with manually crafted templates

To select the optimal LLM, we compared the accuracy of GPT-4 and Llama 2 on Dataset I–S by providing the LLMs with manually crafted task descriptions and guidance.

#### Prompt improvement

##### Prompt augmentation

We explored prompt augmentation to determine if including five examples (five-shot prompting) enhances performance. We adopted five-shot prompting due to the maximum token limitation of GPT-4. Since the selection of examples for few-shot prompting can significantly affect model performance,^32^^,^^34^ we tested four different strategies, including both hard prompting and RAG. To select the best strategy, we chose examples from Dataset I-A and evaluated model performance on Dataset I–S. The four example selection strategies were: 1) Hard Prompting–Random Selection: This strategy involves randomly selecting five samples. 2) Hard Prompting–Targeted Selection: We selected examples where the model had previously performed poorly on Dataset I-A, aiming to directly address its weaknesses. 3) Hard Prompting–K-Means Clustering-Aided Selection: This strategy involves selecting five samples from that are the centres of five clusters generated by k-means clustering. We utilised OpenAI's embedding model, *text-embedding-ada-002*,^34^ as features to ensure the examples are diverse and representative, which could be crucial for performance improvement. 4) RAG—dynamic five-shot: For each case in Dataset I–S, we automatically identified the top five most similar samples from Dataset I-A using OpenAI's embedding model, *text-embedding-ada-002,*^34^ based on the k-nearest-neighbours algorithm. This process enabled us to provide the LLM with five samples that most closely resemble the current case, thereby guiding its decision-making.

##### Error analysis-based instructions

We tested whether incorporating error analysis-based instruction into the prompt could improve performance.^33^ To achieve this, we first conducted an error analysis of the LLM on Dataset I–S. Subsequently, we added a paragraph describing common errors that the LLM made and instructed it to pay attention to those errors when generating its response.

### Baseline machine learning models

We compared the performance of the LLM with two baseline machine learning models developed from our previous study: XGBoost^35^ and a four-layer attention-based deep neural network (DNN),^7^^,^^36^ which incorporated elements of a convolutional neural network, a bidirectional long-short term memory (LSTM) network, and an attention model. Baseline models’ hyperparameters were optimised using grid-search and 5-fold cross-validation on Dataset I. These two models were the top performers compared to other traditional models in identifying cognitive decline in clinical notes.^19^

### Ensemble model

Finally, we investigated whether an ensemble model that combines predictions from both the LLM and traditional machine learning models could achieve better performance. The ensemble learning, which involves combining several different predictions from various models to formulate the final prediction, has proven to be an effective approach for enhancing performance.^37^^,^^38^ To create the ensemble model, we determined the final label using a majority vote method. Specifically, if two or more models (the LLM, the attention-based DNN, and XGBoost) identified cognitive decline, the final label was assigned as cognitive decline; otherwise, it was labelled as no cognitive decline. The high diversity of the models included may enable the ensemble to correct errors made by individual models.^39^

### Model evaluation

We evaluated and compared the selected LLM, traditional models and the ensemble model on Dataset II using standard metrics: $precision/PPV=\frac{true\;positive}{true\;positive+false\;positive}$, $recall/sensitivity=\frac{true\;positive}{true\;positive+false\;negative}$, $f1-score=\frac{2\times precision\times recall}{precision+recall}$, $specificity=\frac{true\;negative}{true\;negative+false\;positive}$, and $NPV=\frac{true\;negative}{true\;negative+false\;negative}$. We used 0.5 as the cutoff point for calculating precision, recall, and F1 score for the baseline models. Additionally, we employed bootstrap resampling on Dataset II to obtain confidence intervals for all metrics and used paired t-tests for statistical analysis, a widely-used and effective approach for model comparison.^40^

### Interpretation

Regarding interpretation, we listed keywords from the LLM's output that appeared more frequently than the average appearance time plus two standard deviations. We also identified keywords whose deep learning attention weights exceeded the mean weights by more than two standard deviations within individual sections, and keywords with an XGBoost information gain higher than the average value plus two standard deviations. Additionally, we included expert-curated keywords developed in our previous study as a reference.^19^

### Error analysis

We conducted two levels of error analyses. The first analysis assessed the selected LLM using various prompting strategies, including zero-shot, the best few-shot method, and the prompt with error analysis-based instructions. The second analysis evaluated the best-performing LLM with its optimal prompt, alongside the attention-based DNN and XGBoost. Errors from each model, encompassing both positive and negative cases, were analysed and discussed by two biomedical informaticians and a physician. A Venn diagram was used to quantify and visualise the unique and overlapping errors made by each model.

### Role of funders

NIH-NIA R44AG081006: This grant provided financial support for the article processing charges, the procurement of computational resources necessary for the study and funded researchers to conduct the research. NIH-NLM 1R01LM014239 and NIH-NIA R01AG080429: These grants supported the researchers involved in completing the study.

The funding sources had no role in the design of the study, data collection, analysis, interpretation, writing of the manuscript, or the decision to submit it for publication. The authors were not paid by any pharmaceutical company or agency to write this article. As the corresponding author, I confirm that all authors had full access to all data in the study and accept responsibility for the decision to submit the manuscript for publication.

## Results

A total of 2166 distinct patients were included. Dataset characteristics are illustrated in Table 2. Dataset I, consisting of 4949 keyword-filtered sections from 1969 patients (1046 [53.1%] women; mean [SD] age, 76.0 [13.3] years), was used to train two baseline models. For prompt development and LLM selection, 200 random samples from Dataset I (Dataset I-S) were used for performance assessment, while the remaining samples (Dataset I-A) were utilised for sample selection in prompt augmentation. Dataset II, which includes 1996 random sections not subjected to keyword filtering from 1161 patients (619 [53.3%] women; mean [SD] age, 76.5 [10.2] years), served for final testing. The average length of the Dataset I sections was 850 characters (range: 26–9393), and that of the Dataset II sections was 464 characters (range: 26–14740). Dataset I contained 29.4% positive cases and Dataset II contained 3.5% positive cases. In addition, the mean time to diagnosis in Dataset I was 1.5 years with a standard deviation (SD) of 1.4 years, while in Dataset II, the mean was 1.9 years with an SD of 1.4 years.

**Table 2:** Dataset characteristics.

Dataset	Description	Mean (range), characters	Positive Rate
Dataset I	4949 note sections filtered with cognitive decline-related keywords	850 (26–9323)	29.4%
Dataset I-A	A random subset of Dataset I, containing 4749 samples	848.8 (26–9323)	29.6%
Dataset I–S	A random subset of Dataset I, comprising 200 samples that do not overlap with Dataset I-A	870.7 (34–8353)	23.5%
Dataset II	1996 random note sections without keyword filtering	464 (26–14740)	3.5%

### LLM selection and prompt selection

**Fig. 2:**
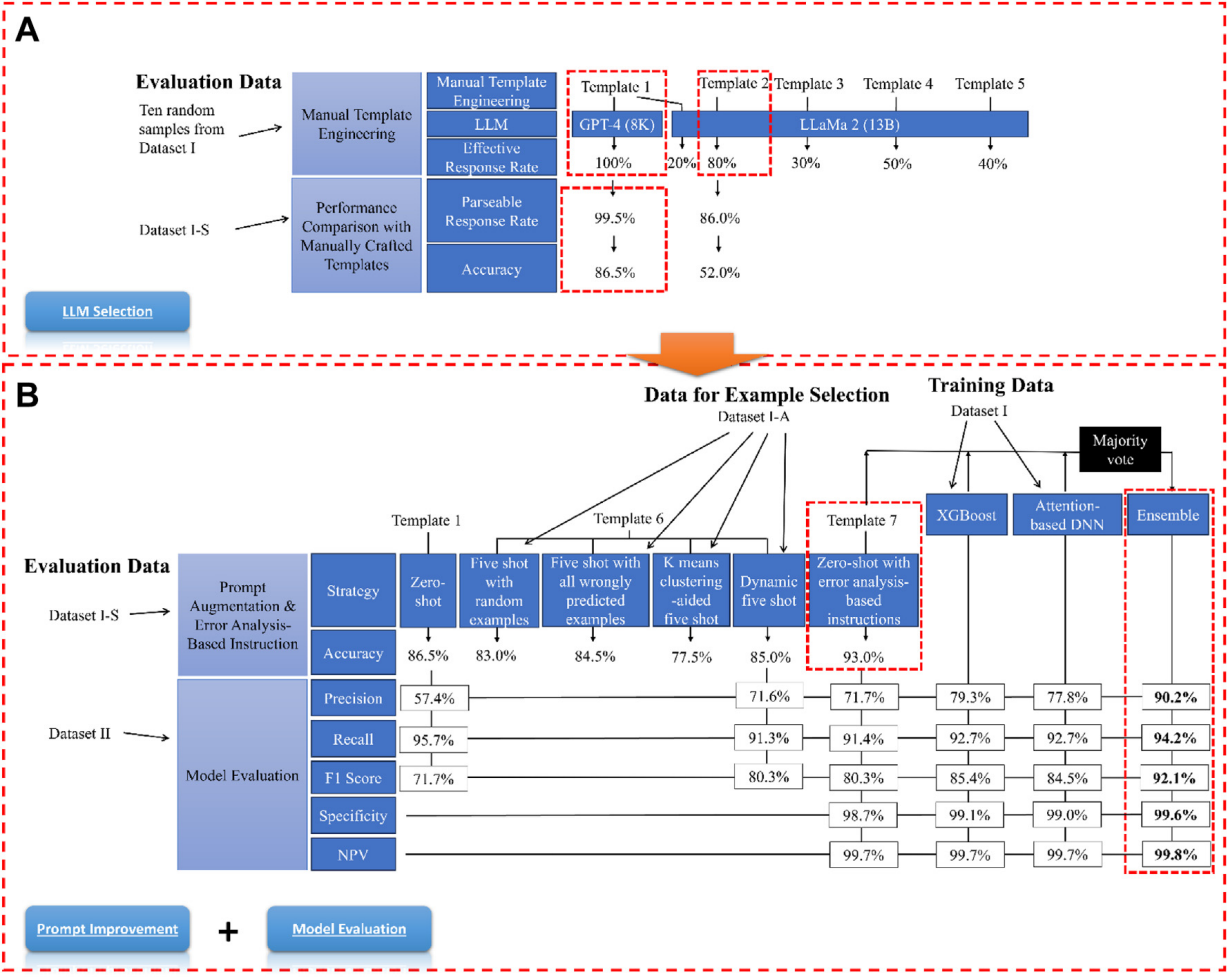
LLM Selection and Model Evaluation Results Summary. Part (A) highlights the LLM selection results. During the prompt template selection, Template 1 was selected for the GPT-4 model due to a 100% effective response rate; Template 2 was selected for the Llama 2 model as the effective response rate (80%) did not improve after three tuning attempts. Subsequently, we compared the two combinations with 200 samples from Dataset I-S and found that GPT-4 and Template 1 combination achieved significantly better accuracy (86.0%). Part (B) of the figure shows prompt improvement and final model evaluation results. This figure only shows averaged values for model evaluation, detailed confidence interval information is illustrated in Supplementary Table S9. We discovered that five-shot prompting did not lead to improved performance; however, adding error analysis-based instructions (i.e., GPT-4 and Template 7 combination) increased the accuracy to 93% on Dataset I–S. Consequently, we opted to use Template 7 as the prompt template and GPT-4 as the LLM. In tests, we evaluated the performance of the XGBoost, the attention-based DNN, and the LLM. We found that XGBoost performed the best: precision—79.3%, recall—92.7%, F1 score—85.4%, specificity—99.1%, NPV: 99.7%. Notably, after combining the three models using a majority vote, the ensemble model demonstrated significantly improved performance: precision—90.2% (an 10.9% improvement), recall—94.2% (a 1.5% improvement), F1 score—92.1% (a 6.7% improvement), specificity—99.6% (a 0.5% improvement), NPV—99.8% (0.1% improvement).

The effective response rate varied for each LLM using five different prompt templates (Fig. 2A). For GPT-4, Template 1, which includes a task description section and additional task guidance section as shown in Supplementary Figure S1, achieved a 100% effective response rate. Llama 2 achieved its highest effectiveness at 80% when using Template 2, which only includes the task description section. GPT-4 and Llama 2, with their most effective prompts, achieved accuracies of 86.5% and 52.0% respectively on Dataset I-S. We therefore chose GPT-4 for subsequent analysis.

Prompt improvement results on Dataset I-S show that the best prompt augmentation approach (Template 6) was RAG—dynamic five-shot, which had an 85% accuracy. However, adding error analysis-based instructions (Template 7) surpassed this, reaching an accuracy of 93%. Therefore, we decided to adopt error analysis-based instructions as our prompting strategy for subsequent analyses.

### Performance evaluation

As shown in Fig. 2B and Supplementary Table S9, GPT-4 achieved a precision of 71.7% [95% CI: 61.7%–80.6%], recall of 91.4% [95% CI: 84.1%–97.2%], F1 score of 80.3% [95% CI: 72.6%–86.7%], specificity of 98.7% [95% CI: 98.1%–99.2%], and NPV of 99.7% [95% CI: 99.4%–99.9%]. Optimized hyperparameters for the attention-based DNN and XGBoost models are detailed in Supplementary Table S8. The attention-based DNN achieved a precision of 77.8% [95% CI: 68.4%–86.3%], recall of 92.7% [95% CI: 85.9%–98.3%], F1 score of 84.5% [95% CI: 77.9%–90.5%], specificity of 99.0% [95% CI: 98.6%–99.5%], and NPV of 99.7% [95% CI: 99.5%–99.9%]. The XGBoost model achieved a precision of 79.3% [95% CI: 70.4%–87.7%], recall of 92.7% [95% CI: 86.3%–98.3%], F1 score of 85.4% [95% CI: 79.1%–91.3%], specificity of 99.1% [95% CI: 98.7%–99.5%], and NPV of 99.7% [95% CI: 99.5%–99.9%].

Notably, the ensemble model significantly enhanced overall performance, achieving a precision of 90.2% [95% CI: 81.9%–96.8%], recall of 94.2% [95% CI: 87.9%–98.7%], F1 score of 92.1% [95% CI: 86.8%–96.4%], specificity of 99.6% [95% CI: 99.3%–99.9%], and NPV of 99.8% [95% CI: 99.6%–99.9%]. Statistical analysis confirmed that the ensemble model outperformed all individual models across all evaluation metrics with statistical significance (p < 0.01). Furthermore, when tested on notes from patients present only in Dataset II, the ensemble model achieved 100% accuracy, outperforming all individual models (Supplementary Table S10).

### Interpretation

Table 1 contains keywords identified through expert curation and exported by GPT-4, the attention-based DNN, and XGBoost. These keywords encompass a range of topics, including memory-related issues such as recall and forgetfulness, cognitive impairments, and dementia, with terms like “dementia” and "Alzheimer's." They also cover evaluation and assessment methods, referencing tools like the MoCA and MMSE. Compared to traditional AI models and expert-selected keywords, GPT-4 highlighted specific treatment options, notably “Aricept” and “donepezil,” (Supplementary Table S9) which are important in managing dementia and Alzheimer's disease. Furthermore, GPT-4 explicitly identified specific diagnoses or conditions more than other models, with terms such as “mild neurocognitive disorder,” “major neurocognitive disorder,” and “vascular dementia.” Additionally, GPT-4 exported keywords regarding the emotional and psychological effects of cognitive disorders, such as “anxiety,” thus addressing aspects sometimes overlooked by other models.

### Error analysis

**Fig. 3:**
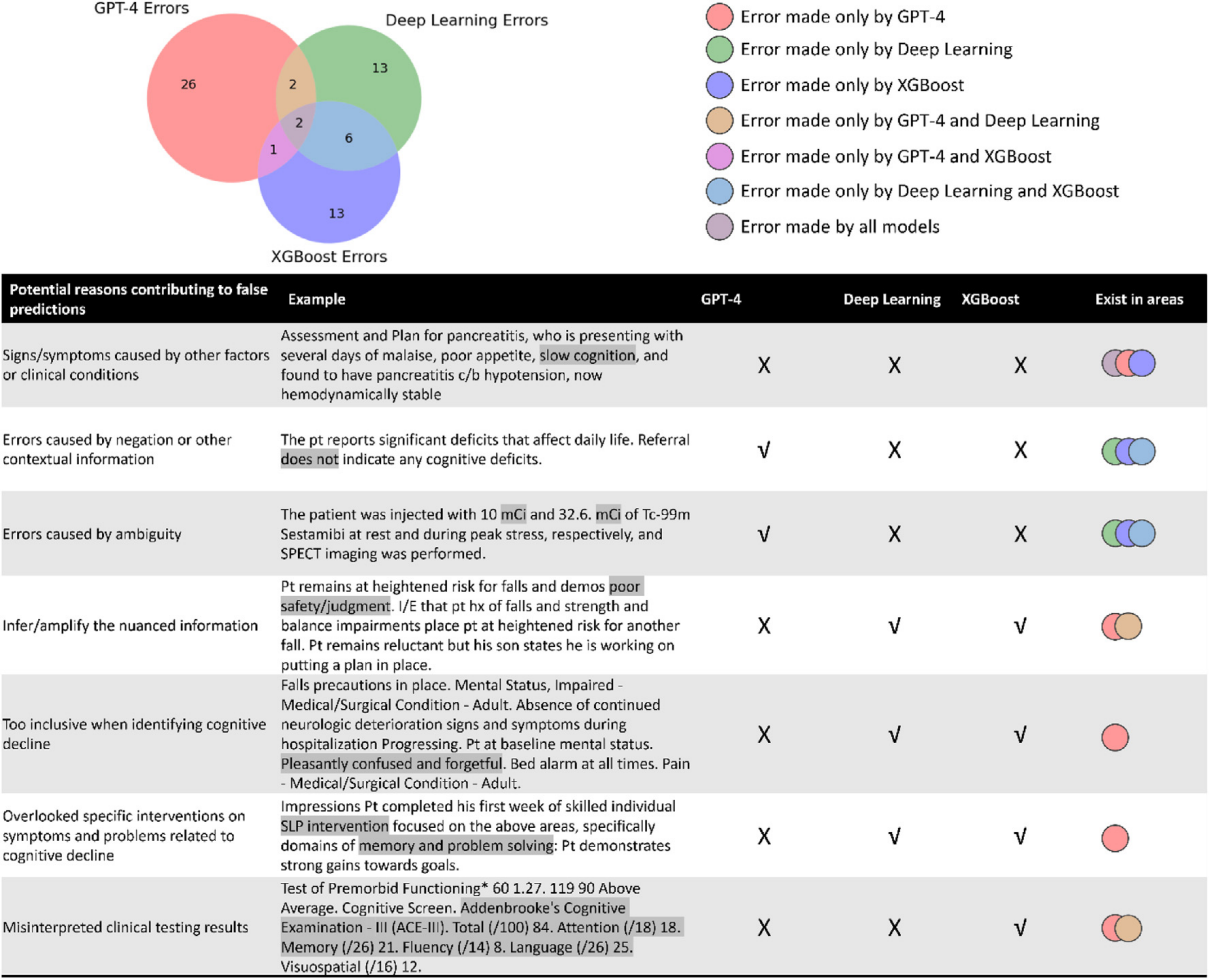
Venn Diagram Highlighting Unique and Overlapping Mistakes Made by Different Models. √: correct prediction; X: incorrect prediction. Some important findings include: 1) All models were susceptible to misinterpreting signs or symptoms as indicative of unrelated clinical conditions. 2) GPT-4 excelled in handling ambiguous terms and interpreting nuanced information, challenges that traditional AI frequently encounters. 3) Unlike traditional models, GPT-4 handles negations and contextual details more efficiently. 4) However, GPT-4 could sometimes overinterpret nuanced information or be overly conservative, failing to recognise whether a patient has cognitive decline despite strong evidence. 5) GPT-4 might also overlook certain medical domain knowledge, such as treatments or visits related to cognitive decline. 6) Both GPT-4 and attention-based DNNs occasionally misread clinical testing results, highlighting an opportunity for further improvement.

As illustrated in Supplementary Figure S2, when using different prompting strategies with GPT-4, some errors may be mitigated, while new ones could emerge that were not previously observed. Notably, adding error analysis-based instructions to the prompt yielded the best performance, with only 31 wrongly predicted cases in Dataset II. In contrast, the error profiles of GPT-4, attention-based DNN, and XGBoost exhibited much higher diversity (Fig. 3). We found that 63 cases were wrongly predicted by one or more models. GPT-4 accounted for 31 incorrect predictions, the attention-based DNN made 23 wrong predictions, and XGBoost was responsible for 22 incorrect predictions. However, only 2 (3.2%) cases were wrongly predicted by all models. Four errors were common between GPT-4 and the attention-based DNN, three were common between GPT-4 and XGBoost, and eight were shared between the attention-based DNN and XGBoost.

All models were susceptible to misinterpreting signs or symptoms as indicative of unrelated clinical conditions. GPT-4 excelled in handling ambiguous terms and interpreting nuanced information, a frequent challenge for traditional AI. Unlike traditional AI, GPT-4 was not confused by negations and contextual details. However, it could sometimes overinterpret nuanced information or be overly conservative, failing to recognise cognitive decline despite strong evidence. It might also overlook underlying causes of clinical events like treatments or visits related to cognitive decline. Both GPT-4 and attention-based DNNs occasionally misread clinical testing results.

## Discussion

Recently, LLMs have demonstrated remarkable performance on various NLP tasks, yet their ability to analyse clinical notes from EHR data remains underexplored, partly due to data privacy concerns. In this study, we established HIPAA-compliant secure environments for LLMs and used cognitive decline identification as a use case to test LLMs' capabilities in clinical note classification, thereby enhancing diagnostic tasks. Our contributions are threefold: 1) We set up a secure cloud environment for GPT-4 and tested its ability to identify cognitive decline from clinical notes in EHR data; 2) We introduced a method for implementing NLP models for cognitive decline identification, achieving state-of-the-art performance with a significant lead over existing methods; 3) We discovered that although existing LLMs may not outperform traditional AI methods trained on a local medical dataset, their error profile differs distinctly, underscoring the significant potential of combining LLM with traditional AI models. The end goal of our proposed approach is to employ cutting-edge AI techniques to enable physicians to detect early and flag patients with “high” risk on the EHR system accurately so that they can start acting on preventive treatments.

Our research demonstrated that prompt engineering using error analysis-based instructions significantly enhanced performance compared to zero-shot and prompt augmentation approaches, as it directly targeted the LLM's weaknesses. Nevertheless, the LLM did not surpass traditional AI in identifying cognitive decline, primarily because it was not specifically trained for this task.^9^^,^^41^ While the LLM can generate a range of responses, it is prone to producing plausible but incorrect hallucinations. Nonetheless, it is valuable for its ability to operate without task-specific training, thereby complementing traditional AI, which requires specific training but often does not suffer from hallucinations.^42^ In terms of interpretation, the LLM identified keywords overlooked by experts and traditional AI models, such as medications related to cognitive decline. Notably, we observed LLM identified medications for AD/ADRD prior to the coding of MCI, indicating LLMs could function as a decision-support tool, flagging notes that imply cognitive decline and alerting clinicians to investigate further, even if the diagnosis has not been explicitly established in the records. Error analysis revealed that the LLM demonstrated superior handling of ambiguous or contextually complex information due to its transformer architecture.^3^^,^^4^ However, LLMs misinterpreted or overlooked certain domain-specific medical tests and treatments. Future research should explore the integration of the LLM with smaller, localised models and knowledge bases to enhance performance on specific tasks.

Our proposed method and results hold the potential for meaningful integration into clinical practice. By incorporating accurate predictions derived from clinical note sections, we can enhance patient-level diagnosis through the identification of trends and patterns indicative of cognitive decline, as observed across sequential patient notes over time. This approach allows for a dynamic and continuous assessment of a patient's cognitive trajectory, facilitating early detection of subtle changes that may precede a formal diagnosis. Moreover, our method can be embedded within a clinical decision support system, which would synergise predictions from note sections with other relevant patient data, such as magnetic resonance imaging (MRI) results, laboratory findings, neurologic tests, and genetic information. This multimodal approach offers clinicians a more holistic and precise evaluation of a patient's cognitive status, enabling the detection of cognitive decline at earlier stages and guiding more timely and informed clinical decisions. For instance, the system could prompt clinicians to recommend specific cognitive assessments, adjust treatment plans, or initiate early interventions, ultimately improving patient outcomes through personalised and proactive care.

Although our study has several strengths, such as employing LLMs on unstructured EHR data for detecting cognitive decline, the results should be interpreted considering certain limitations. The LLMs used may not represent the most recent advancements (e.g., the recently released Llama 3 model) due to the rapid evolution of LLM technologies. While utilising LLMs with a larger number of parameters (e.g., Llama 2–70 billion) may lead to better performance, this improvement comes with trade-offs, including higher computational demands and greater memory needs, posing challenges due to resource constraints. Additionally, our data are record-based and not patient-based (i.e., longitudinal), thus, the developed model may struggle to distinguish between reversible and progressive cognitive decline, and it remains unclear if patients recovered later based solely on a note from one time point. Patients obtained from our patient identification method (ICD-10 of G31.84) might not be confirmed MCI cases. Therefore, developing an LLM-based early warning system for cognitive decline using longitudinal data would be a valuable direction for future research. Additionally, exploring the relationship between predictive accuracy and time to diagnosis would be an interesting direction for future research. Furthermore, our training data (Dataset I) and testing data (Dataset II) were not split based on patients. Although the evaluation of the ensemble model on 267 notes from 196 patients exclusively present in Dataset II demonstrated 100% accuracy and outperformed all individual models, a larger testing dataset that includes patients not found in Dataset I is needed in future studies to provide a more robust evaluation.

## Conclusion

This study utilised LLMs within HIPAA-compliant cloud environments, leveraging real EHR notes to detect cognitive decline. Our findings indicate that LLMs and traditional models exhibit diverse error profiles. The ensemble of LLMs and locally trained machine learning models on EHR data was found to be complementary, significantly enhancing performance and improving diagnostic accuracy. Future research could investigate methods for incorporating domain-specific medical knowledge and data to enhance the capabilities of LLMs in healthcare-related tasks.

## Contributors

Conceptualisation and design: XD, LZ; HIPAA-compliant computational environment setup: XD, JMP, FC, BL, LZ; Data preparation—cognitive decline case annotation: JNL, LW, LZ; Data preparation—error analysis: XD, JNL, YWC, LZ; Programming and AI model development: XD, JNL; Results interpretation: XD, JNL, YWC, LW, GAM, SKM, SD, HP, BL, QW, XW, JW, HD, FJM, JD, DWB, LZ; Original draft preparation including tables and figures: XD; Critical revision of the manuscript for important intellectual content: XD, JNL, LZ; Funding acquisition: JD, LZ; Supervision: LZ.

Authors that have accessed and verified underlying data: XD, YWC, LZ.

All authors read and approved the final version of the manuscript.

## Data sharing statement

A study protocol was not prepared or registered. Patient data is not available given their potentially identifiable nature. Additional information and resources regarding the study are available upon reasonable request to authors: Xinsong Du (xidu1@bwh.harvard.edu), John Novoa-Laurentiev (jnovoa-laurentiev@bwh.harvard.edu), Li Zhou (lzhou@bwh.harvard.edu).

## Patients and public involvement

There is no patient and public involvement during the design, conduct, reporting, interpretation, or dissemination of the study.

## Declaration of interests

None.
